# Dietary trends among young adults during the COVID-19 lockdown: socioeconomic and gender disparities

**DOI:** 10.1186/s40795-023-00759-0

**Published:** 2023-09-25

**Authors:** Jennifer Parker, Simranpreet Kaur, John Marlo Medalla, Anairobi Imbert-Sanchez, Jeanette Bautista

**Affiliations:** 1 Pennsylvania State University, 2809 Saucon Valley Road, Center Valley, Pa, 18034 USA; 2 Bachelor of Science Student in Biobehavioral Health, Pennsylvania State University, 2809 Saucon Valley Road, Center Valley, Pa, 18034 USA

**Keywords:** COVID-19, Diet, Health, Inequities, Young adults

## Abstract

**Background:**

Healthy eating is vital to well-being and during the COVID-19 pandemic, it was especially important for boosting immunity and protecting against viral infections. Yet, by many accounts, keeping a nutritious diet was a casualty of the pandemic rather than a means to fight it. Young adults experienced disproportionate pandemic-related disruptions during a formative stage of development while little is still known about dietary outcomes.

**Methods:**

We employed a cross-sectional design to examine dietary disparities targeting young adults (ages 18–28) during the COVID-19 lockdown period. Participants (*N* = 254) responded to a 15–20-min online survey with questions related to food composition and sources of food, perceptions of healthy eating, weight change, physical activity, and food insecurity. Comparisons were made by household income and gender. Multiple regression analyses were conducted to investigate factors that predicted perceptions of healthy eating behaviors while controlling for other sociodemographic factors.

**Results:**

A clear overall trend toward unhealthy behaviors was found while positive changes were also identified. Consumption of junk food significantly increased (+ 3%), 40% gained weight, a third were less active, and 5–8% were food insecure on a regular basis. Meanwhile, eating food from restaurants declined and, for some, home-based cooking increased. Lower income participants were overly represented in unhealthy changes and higher income participants were disproportionately represented in healthy changes. Males reported more changes in dietary composition while females reported more fluctuation in weight. Reduced activity, weight gain, and food insecurity predicted unhealthy eating behaviors. Living with friend(s)/roommate(s) predicted healthier eating, but only among lower income participants.

**Conclusions:**

It is recommended that pandemic minded public health interventions account for negative dietary trends with particular attention to low-income young adults. Solutions should be geared toward reshaping fiscal, social and physical environments, rather than relying solely on behavioral interventions.

## Background

Healthy eating is vital to health and well-being and during the COVID-19 pandemic it was especially important. A balanced diet, rich in nutrients, is known to boost immunity, help protect against viral infections, and preserve long-term well-being [1–6]. Yet, by many accounts, a healthy diet was a casualty of the pandemic, rather than a means to fight it. In the early stage of the pandemic, a global economic model predicted disrupted food supply chains, price destabilization, hampered food access, and a shift away from nutrient-rich foods, such as fruit, meat, eggs, and dairy. This was expected to result in an increase in consumption deficiencies due to a lack of micronutrient content while intensifying already existing cases of undernourishment, especially among the poverty stricken [7, 8].

While global organizations such as the International Food Policy Research Institute, the World Food Programme, and UNICEF, called attention to the devastating impacts on the world’s most impoverished regions such as in India and Sub-Saharan Africa [8], the circumstances were also dire in the wealthiest countries, including in the United States. The National Bureau of Economic Research declared the COVID-19 pandemic to have caused the worst American recession since the Great Depression [9] with growing numbers of people suffering joblessness, financial strains, and lack of access to nutrient rich food [10].

Lifestyle behaviors during lockdown conditions were found to reinforce these disturbing trends [11]. Studies found that more time spent at home promoted hypercaloric intake with larger meal sizes and increased frequency of snacking [12, 13]. One study found this to be especially true for females whose energy intake was about 20% greater during the pandemic [12]. Other studies linked poor eating habits to depressed moods and anxious feelings [4, 14–16]. Concerning issues with inactivity and weight control were also tied to the pandemic [13–15, 17–20].

But not all depictions of the pandemic’s impact on diet were negative [21–23]. There were plenty of rosy accounts of families making the best of times hunkered down together, cooking nutritious recipes, and gathering around the dinner table. One study found that with people staying at home and a decline in “eating out,” there was more consumption of home cooked meals, less fried foods, and with it, positive dietary changes and prevention of obesity [24]. Private industry studies reported more cooking, use of recipes, and confidence in healthy meal preparation [25, 26]. Narrative pieces in *Gastronomica* stressed a movement toward “creative living” and authenticity where home cooking was reconceptualized as a means for gaining a sense of control and agency during a time when everything else seemed out of control [27, 28]. In these depictions, COVID-19 seemed to have reclaimed the kitchen, food, and cooking, as central to dietary health and wellness.

Unfortunately, in this abundant literature little attention has been paid to group level socioeconomic differences. This is important given the inequalities brought on by the pandemic and especially the evidence of socioeconomic disparities in COVID-19 related health outcomes [29–31]. In one study, socioeconomic advantage was associated with lower odds of having a poor appetite while in lockdown [32]. Another study found that food insecurity was related to less cooking at home among low-income adults [33]. Research that focused on children and adolescents in Brazil found that lower-class families in home confinement and those from the Northeast region consumed a less healthy diet, with less fruits, juices, vegetables, and beans, than their more privileged counterparts [34].

Considerably more scholarly research has addressed gender differences in dietary concerns during the COVID-19 pandemic given sociocultural norms around household responsibilities and caregiving, and pre-existing gender-based vulnerabilities to stress, depression, and other diseases [35–37]. But the results of these studies of the pandemic’s impact on gender have been mixed. On the one hand, an abundant of research has shown that women’s dietary health suffered more than men’s during the pandemic [21, 38–41]. Several studies, for instance, have shown that women were more prone to stress and/or depression during the pandemic, which in turn, impacted dietary behaviors and overall health [21, 38, 42, 43]. A study based in Southern California, that also looked at sexual minorities, found that heterosexual women and sexual minorities were more likely to engage in adverse eating behaviors and self-harm than heterosexual men [39]. Some studies give evidence of women’s greater tendency to have deviated from their own dietary customs during the pandemic such as women in Italy who strayed from their Mediterranean diet [40] and women in Pakistan who trended away from their normally nutritious and diverse diet [41]. On the other hand, other studies such as one based in Spain [44], found that home confinement led to women developing better, not worse, dietary habits than men. Likewise, an Australian study found that it was men whose dietary habits became unhealthier during the pandemic, including their increased consumption of alcohol [45].

The current study contributes to this literature with a focus on the dietary practices of young adults, a largely overlooked demographic in studies on COVID-19’s impact on diet. The exception are studies that target college students and that therefore miss representation of a broader swath of the young adult population [46–50]. Young adults are an important demographic to consider since they experienced some of the most severe pandemic-related disruptions during a formative stage of development, a stage that is known to have profound implications on career paths, life-long economic security, and future bodily health [51]. During the pandemic young adults experienced disproportionate job losses [52, 53], had social lives abruptly curtailed at a time of life when peer groups are especially important [54], and many were uprooted from college environments. These changes are even more critical considering that before the pandemic, young adults were already experiencing alarming rates of depression, stress, and suicide [55, 56] which may have set them up to be exponentially vulnerable to COVID-19 related health problems.

The dietary practices of young adults during the pandemic could have either helped or hindered the vast challenges they faced at a time when a healthy diet was essential to well-being. This study specifically asked, how did the COVID-19 pandemic affect the dietary habits of young adults in the United States and how did the effects vary by socioeconomic group and by gender? Did dietary practices of young adults reflect the American class divide that was exacerbated during the pandemic? Did young men and women vary in their dietary behaviors and perceptions in ways that need our attention? Or was diet an aspect of life that young people experienced on an even playing field during the pandemic? Dietary practices on a broad range of measures were investigated. The intention was to contribute knowledge that could be useful for developing socioeconomically and gender sensitive policies aimed at mitigating harmful long-term outcomes of the COVID-19 pandemic while preventing nutritional inequities during future public health crises.

## Methods

### Participants

This study employed a cross-sectional design. Inclusionary criteria included being 18 to 28 years of age and living in the United States. An anonymous questionnaire was developed specifically for this study and was distributed through the Qualtrics platform online through relevant social media groups on Facebook and Instagram. Purposive sampling technique was used. It was also distributed through a student email listserve of the university where the study took place. The questionnaire was available for a period of 20 days from November 10 to November 30, 2020. This online method was considered effective because it allowed for dissemination at a time when face-to-face distribution was restricted due to the pandemic and when internet usage was up due to home confinement measures [57]. The study received approval from the Institutional Review Board of the university where the study took place. All methods, including procedures for obtaining consent were applied in accordance with the Institutional Review Board’s guidelines and regulations. Prior to participating in the study respondents were informed of the study’s intentions and the voluntary nature of their participation. They also verified being at least 18 years of age.

A total of 361 people attempted the survey. Participants who did not meet the inclusionary criteria and/or did not complete the survey were excluded. The result was a sample size of (*N* = 254) (Table 1). Lower-income participants made up 62% and higher-income participants comprised 38% of the sample. Most were female (71%). Males and females were distributed evenly across income categories (*P* = 0.559). The mean age of participants was 20.93 + 2.33 (SD). Most (70%) reported living with a parent(s), more than half (56%) lived with other family members, and some (18%) lived with a friend(s) and/or roommate(s) during the early stage of the pandemic. The biggest race/ethnic group self-reported as white (40%) followed by Latino/Hispanic (23%), Asian (16%), multiracial (13%), and black/African American (4%). The majority (88%) reported living in the Northeast region of the United States. Slightly more than half were current students (60%) and about two-thirds (67%) were currently employed in a paid job.

**Table 1:** Sociodemographic characteristics of the study population (*N*= 254)

Characteristics	n (%)
**Age (years) Mean (SD)**	
**Gender**	
Male	71 (28.0)
Female	180 (70.9)
Non-binary or other	2 (.8)
**Household income**	
Higher-income	96 (37.9)
Lower-income	157 (62.1)
**Race/ethnicity**	
White	100 (40.3)
Black/African American	11 (4.4)
Latinx	58 (23.4)
Asian	39 (15.7)
Multiracial	31 (12.5)
Other	9 (3.6)
**Student status**	
Current student	152 (59.8)
Not a current student	102 (40.2)
**Employment status**	
Currently employed	171 (67.3)
Not employed	83 (32.7)
**Who lived with**	
Parents	178 (70.4)
Other family members	143 (56.3)
Friends/roommates	45 (17.7)

### Measures

#### Composition of the diet and sources of food

Participants were asked to report, retrospectively, on the composition of their diet and the sources of their food for two periods–before the pandemic and during the first few months of the pandemic. Composition of the diet was measured by “constant sum” questions to assess how the overall distribution of the diet across five food categories changed. Food groups were based on USDA (U.S. Department of Agriculture) and PCRM (Physicians Committee for Responsible Medicine) guidelines for a healthy diet and included meats, dairy, grains, and fruits/vegetables. The authors added the category “junk food.” Sources of food were also measured by “constant sum” questions to assess changes in where food came from across five different sources. These included restaurants, fast food establishments, pre-prepared food, home cooked food from scratch, and junk food.

#### Healthy eating behaviors

Healthy Eating behaviors were measured based on an eight-item Healthy Eating Index (HEI) that the authors developed and adapted from previous questionnaires conducted during the COVID-19 pandemic [4, 16, 58, 59]. Participants were asked: “To what extent did the following habits become less healthy or more healthy during the first few months of the COVID-19 pandemic compared to before the pandemic?” Items ranged from overall perception of healthy eating and “indulging in unhealthy foods during boredom or distress” to “sense of hunger and satiety” and “amount/number of meals prepared at home.” Each item was based on a Likert scale (from 1, much less healthy to 5, much more healthy) and analyzed individually, and then, compositely (as a continuous index variable). The intent was to evaluate changes in eating habits from before the pandemic to the first few months of the pandemic in respect to how participants perceived their behaviors. Possible total scores on the composite index ranged from 8 (much less healthy) to 40 (much more healthy). An overall score that was < 24 was considered an overall unhealthy change while a score of > 24 was considered an overall healthy change. The alpha coefficient (Cronbach’s Alpha) for the eight items comprising the HEI was 0.787, suggesting a high internal consistency [60]. The skewness of continuous variables ranged from 0.054 to 1.824 and kurtosis ranged from -0.858 to 6.458, showing robust distributions within acceptable ranges for parametric testing [61, 62]. The alpha coefficient (Cronbach’s Alpha) for the eight items comprising the HEI was 0.787, suggesting a high internal consistency [60]. The skewness of continuous variables ranged from 0.054 to 1.824 and kurtosis ranged from -0.858 to 6.458, showing robust distributions within acceptable ranges for parametric testing [61, 62].

#### Food insecurity

Food insecurity was evaluated based on items adopted from the USDA Measurement of Household Food Security Survey [63] with a focus on evaluating both extent of worry about having enough food, and actually having enough to eat. Respondents replied on a Likert scale from 1(never, always had enough) to 5 (most of the time). In regression analysis, these two scales were combined to form an index variable. The combined scales had a Cronbach’s alpha of 0.821, showing a high degree of reliability.

#### Changes in physical activity and weight

Change in physical activity level was measured on a Likert scale from 1 (much less) to 5 (much more) activity. Change in weight was also measured on a Likert scale from 1 (significant decrease) to 5 (significant increase).

#### Who participants lived with

Participants were asked to report on who they lived with during the early stage of the pandemic including parent(s), other family members, and/or friends/roommates.

#### Core demographic variables

Our core demographic variables were household income and gender because of the evidence in the literature of COVID-19 related vulnerabilities related to low-income and female groups [29, 30]. Household income consisted of two categories, lower-income (< $70,000) and higher-income (≤ $70,000). The divide between income groups is based on median household income in the United States for 2019, the year prior to the pandemic ($68,400) [64]. Students living on their own were requested to report the income of the household where they were declared a dependent. Independent adults were asked to exclude the income of non-related household members. Gender consisted of multiple categories, male, female, non-binary and “other.” Gender based analysis included only the categories of male and female since only three respondents identified as non-binary or “other.” Covariates were included in regression analysis and consisted of age, student status, employment status, and who the participant lived with during the pandemic’s earliest stage.

#### Data analysis

SPSS software (version 27) (SPSS Inc., Chicago, IL, USA) was used for all analysis. Paired sample *t*-tests were conducted to investigate changes in dietary composition and sources of food. Cross tabulations were conducted to test associations between core demographic variables and changes in physical activity levels, weight, food insecurity, and individual items on the HEI index. Independent sample *t*-tests were used to compare mean scores of the HEI by household income and gender. Multiple linear regressions were run to find predictors of the HEI index. Statistical significance was considered at a *P*-value of < 0.05. Multicollinearity was checked with the aim of a VIF (Variance Inflation Factor) less than 5.

## Results

### Changes in dietary consumption

**Fig. 1:**
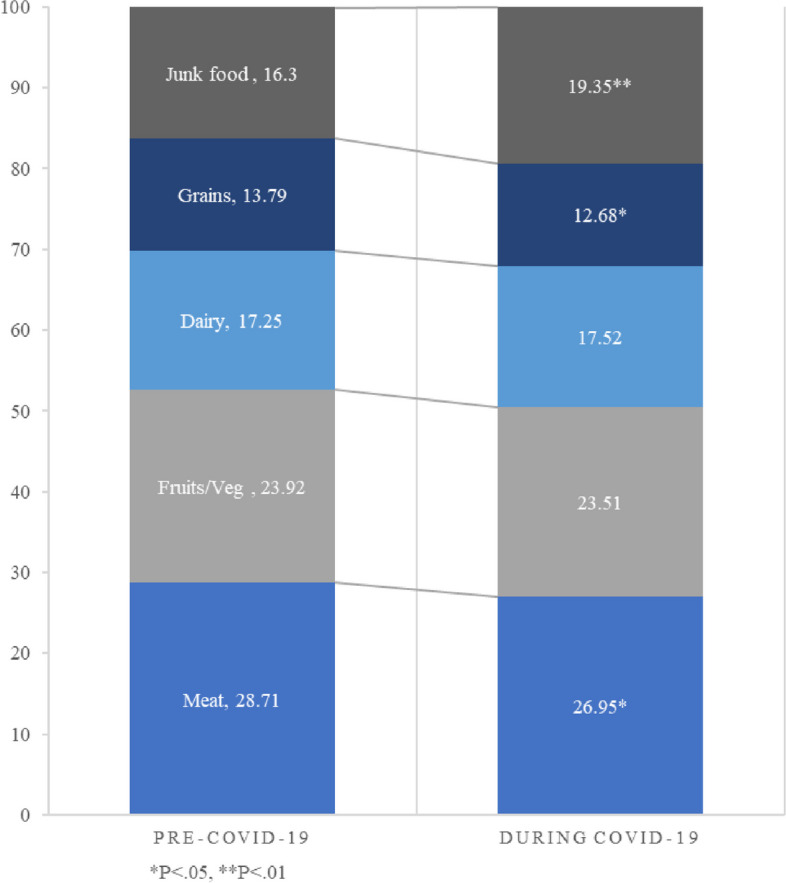
Change in reported dietary composition (% of overall diet)

The biggest overall change in dietary composition was a reported increase in eating junk food, from 16% (SD = 13.06) of the diet before COVID-19 to 19% (SD = 14.61), (*P* = 0.008) during the first few months of the pandemic (Fig. 1). Meanwhile, reported meat consumption declined from (29% (SD = 18.12) to 27% (SD = 16.56), (*P* = 0.039) and reported grain consumption declined from 14% (SD = 11.79) to 13% (SD = 11.73), (*P* = 0.028) (Fig. 1). While every social group reported an increase in eating junk food, the increases were statistically significant only among lower income (*P* = 0.049) and male (*P* = 0.002) participants. Similarly, while every social group reported a decrease in the consumption of grains, the increases were significant only among these same groups–lower-income (*P* = 0.014) and males (*P* = 0.003). The decline in reported meat consumption was found only among higher-income participants (*P* = 0.044) and males (*P* = 0.042) (Table 2).

**Table 2:** Dietary composition and sources of food pre-COVID-19 and during COVID-19 by household income and gender

**Dietary composition**		**Pre-Covid**		**During Covid**		**Change**	***P*-value**
		**M**	**SD**	**M**	**SD**		
Meat	Lower income	29.32	18.89	28.07	16.57	-1.25	.271
	Higher income	27.63	16.91	25.04	16.51	-2.59	.044
	Male	34.32	16.74	30.49	15.56	-3.83	.042
	Female	26.21	17.48	25.68	16.86	-0.53	.542
Fruits/vegetables	Lower income	22.75	12.95	22.11	12.31	-0.64	.578
	Higher income	25.44	17.64	25.36	15.84	-0.08	.961
	Male	20.38	10.96	19.92	10.92	-0.46	.721
	Female	25.63	16.15	25.06	14.91	-0.57	.620
Dairy	Lower-income	17.57	10.64	18.29	11.64	0.72	.386
	Higher-income	16.91	10.00	16.44	10.55	-0.47	.675
	Male	16.66	9.61	16.58	11.23	-0.08	.954
	Female	17.40	10.62	17.76	11.28	0.36	.626
Grains	Lower-income	13.78	11.56	12.05	11.29	-1.73	.014
	Higher-income	13.96	12.19	13.83	12.40	-0.13	.856
	Male	14.86	12.32	12.32	12.33	-2.54	.003
	Female	13.39	11.56	12.70	11.55	-0.69	.261
Junk food	Lower-income	16.55	12.64	19.48	14.36	2.93	.049
	Higher-income	16.06	13.74	19.32	15.04	3.26	.071
	Male	13.69	11.51	20.68	15.78	6.99	.002
	Female	17.37	13.54	18.80	14.24	1.43	.281
**Source of food**							
Restaurants	Lower-income	17.90	16.02	13.90	16.52	-4	.008
	Higher-income	17.44	15.42	11.90	14.95	-5.54	.005
	Male	18.55	18.67	14.44	16.00	-4.11	.050
	Female	17.55	14.53	12.82	15.97	-4.73	.001
Fast food	Lower-income	14.89	13.30	16.58	16.59	1.69	.209
	Higher-income	15.40	13.28	11.77	13.64	-3.63	.013
	Male	14.69	12.65	15.75	15.35	1.06	.543
	Female	15.33	13.49	14.59	15.87	-0.74	.550
Home cooked (pre-prepared)	Lower-income	16.90	16.64	16.57	16.66	-0.33	.778
	Higher-income	16.28	18.54	17.56	19.26	1.28	.556
	Male	21.65	21.30	20.30	20.94	-1.35	.651
	Female	14.68	15.20	15.40	15.99	0.72	.463
Home-cooked (by scratch)	Lower-income	41.08	24.39	40.33	26.81	-0.75	.729
	Higher-income	40.81	26.05	46.41	29.01	5.6	.040
	Male	35.69	23.29	37.03	26.11	1.34	.698
	Female	43.04	25.04	44.66	28.02	1.62	.403
Junk food	Lower-income	9.34	10.32	12.43	10.91	3.09	.002
	Higher-income	10.07	10.92	12.36	12.22	2.29	.115
	Male	9.52	11.69	12.20	10.70	2.68	.037
	Female	9.46	9.88	12.48	11.76	3.02	.004

M, Mean, SD, Standard Deviation. The test statistic is a paired sample *t* test comparing food composition and sources of food from before the pandemic to the first few months of the pandemic. Statistical significance was established at *P* < *.05*

### Changes in sources of food

**Fig. 2:**
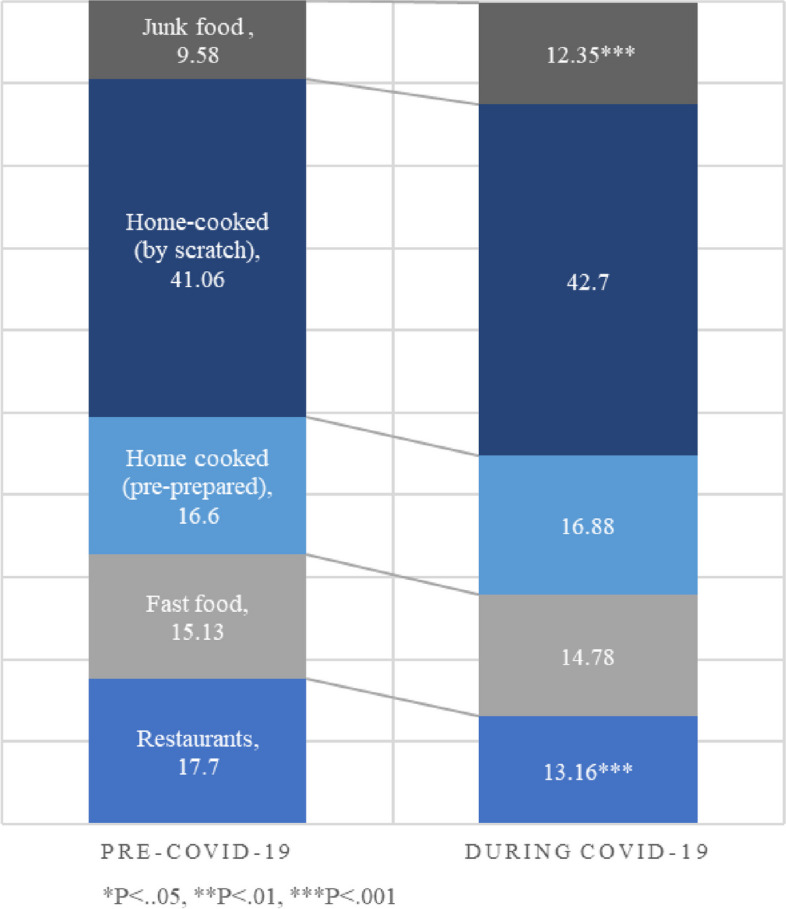
Change in reported food sources (% of overall diet)

Participants reported a decline in getting food from restaurants from 18% (SD = 15.74) to 13% (SD = 15.91), (*P* < 0.001) while junk food sources increased from under 10% (SD = 10.53) before the pandemic to over 12% (SD = 11.41), (*P* < 0.001) during the pandemic (Fig. 2). Both males (*P* = 0.037) and females (*P* = 0.004) reported significant increases in junk food sources. But only lower-income (*P* = 0.002) and not higher-income (*P* = 0.115) participants reported significant increases. Meanwhile, only higher-income respondents reported a significant decline in getting food from fast food places (*P* = 0.013) and a significant increase in eating home-based meals that were cooked from scratch (Table 2).

### Changes in physical activity and weight

Over 40% of participants reported gaining some or a lot of weight while nearly one-in-five (17%) reported losing weight. Lower-income participants (50%) and females (45%) had significantly higher rates of gaining weight than their higher-income (29%) and male (38%) counterparts. Meanwhile, higher-income (22%) and female (20%) participants had higher rates of losing weight compared to lower-income (15%) and male (10%) participants. A third of the participants (33%) reported becoming less active and more than a third (39%) became more active. There were no significant social group differences in reported activity levels (Table 3).

**Table 3:** Comparisons of weight, physical activity, and food insecurity, by household income and gender

			**Household Income**			**Gender**		
**Variable**		**Overall** **n (%)**	**Lower** **n (%)**	**Higher** **n (%)**	***P*-value**	**Male** **n (%)**	**Female** **n (%)**	***P*-value**
Change in weight	Sig. decrease	6 (2.4)	3 (1.9)	3 (3.1)	.024	3 (4.2)	3 (1.7)	.032
	Decrease	38 (15.0)	20 (12.7)	18 (18.8)		4 (5.6)	32 (17.8)	
	No change	102 (40.2)	55 (35.0)	47 (49.0)		37 (52.1)	65 (36.1)	
	Increase	93 (35.6)	67 (42.7)	25 (26.0)		24 (33.8)	68 (37.8)	
	Sig. increase	15 (5.9)	12 (7.6)	3 (3.1)		3 (4.2)	12 (6.7)	
Change in physical activity	Much less	28 (11.0)	17 (10.8)	11 (11.5)	.882	13 (18.3)	13 (7.2)	.061
	Somewhat less	56 (22.0)	38 (24.2)	18 (18.8)		17 (23.9)	38 (21.1)	
	No change	72 (28.3)	44 (28.0)	27 (28.1)		20 (28.2)	52 (28.9)	
	Somewhat more	68 (26.8)	40 (25.5)	28 (29.2)		16 (22.5)	52 (28.9)	
	Much more	30 (11.8)	18 (11.5)	12 (12.5)		5 (7.0)	25 (13.9)	
Food insecurity-worried about next meal	Never, always enough	185 (72.8)	100 (63.7)	84 (87.5)	.001	45 (63.4)	139 (77.2)	.015
	Once or twice	29 (11.4)	23 (14.6)	6 (6.3)		7 (9.9)	21 (11.7)	
	Occasionally	28 (11.0)	24 (15.3)	4 (4.2)		12 (16.9)	15 (8.3)	
	Regularly	7 (2.8)	5 (3.2)	2 (2.1)		3 (4.2)	4 (2.2)	
	Most of the time	5 (2.0)	5 (3.2)	0 (0)		4 (5.6)	1 (.6)	
Food insecurity-Not enough to eat	Never, always enough	159 (62.6)	83 (52.9)	75 (78.1)	.001	39 (54.9)	119 (66.10)	.392
	Once or twice	40 (15.7)	34 (21.7)	6 (6.3)		13 (18.3)	26 (14.4)	
	Occasionally	38 (15.0)	28 (17.8)	10 (10.4)		12 (16.9)	26 (14.4)	
	Regularly	10 (3.9)	7 (4.5)	3 (3.1)		5 (7.0)	5 (2.8)	
	Most of the time	7 (2.8)	5 (3.2)	2 (2.1)		2 (2.8)	4 (2.2)	

The test statistic is *X*^*2*^ (Chi square). Statistical significance was established at *p* < .05

### Food insecurity

Nearly a quarter (22%) reported not having enough to eat at least occasionally and 16% indicated that they worried about where their next meal was coming from at least occasionally during the early part of the pandemic. Lower-income participants reported higher rates of food insecurity on both food security measures, while males reported higher rates than women in respect to worrying about where their next meal would be coming from (Table 3).

### Perceptions of healthy eating

**Fig. 3:**
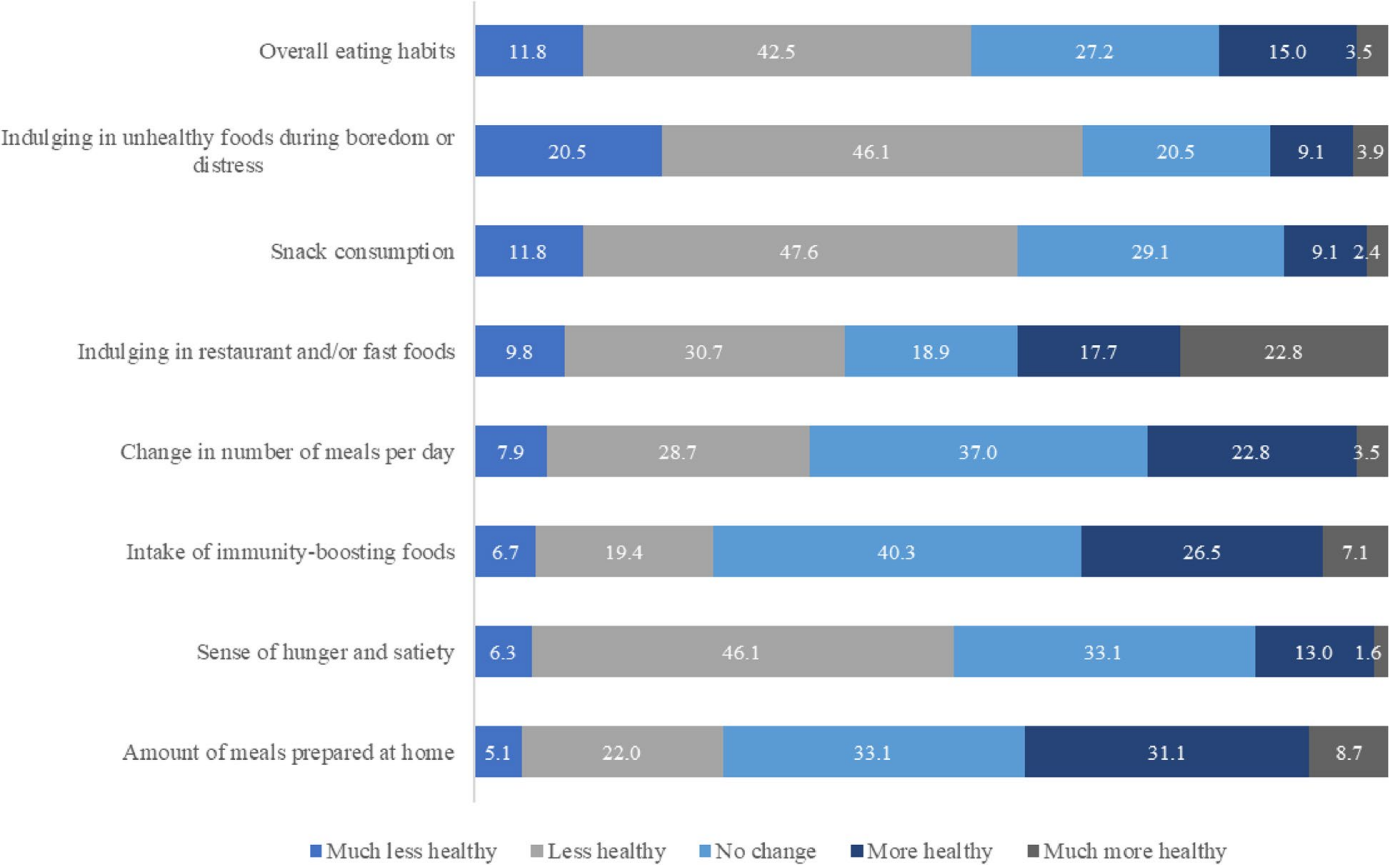
HEI (Healthy Eating Index) items. Responses (by %) on a scale of 1(much less healthy) to 5 (much more healthy)

The mean HEI score was 21.96 (SD = 5.38, range: 8 to 38) which indicates a change toward unhealthier dietary habits during the first few months of the pandemic (Table 4). More than half of the participants reported “less healthy” or “much less healthy” in four out of ten of the individual items that made up the HEI scale (Fig. 3). Lower-income participants had a significantly lower mean HEI score than higher-income participants (21.0 vs. 23.53, *P* < 0.001) and were significantly overrepresented in unhealthy trends in five of the individual items that make up the healthy eating index. There were no significant differences in HEI scores by gender.

**Table 4:** HEI (Health Eating Index) and Individual Items

		**Household Income**			**Gender**		
	**Overall** **M(SD) or n (%)**	**Lower ** **M(SD) or n (%)**	**Higher ** **M(SD) or n (%)**	***P*-value**	**Male** **M(SD) or n (%)**	**Female** **M(SD) or n (%)**	***P*-value**
Healthy Eating index*	21.96 (5.38)	21.0 (4.99)	23.53 (5.66)	<.001	21.96 (5.63)	21.98 (5.34)	.973
**Overall eating habits**							
Much less healthy	30 (11.9)	21 (13.4)	9 (9.4)	.041	7 (9.9)	22 (12.2)	.617
Less healthy	108 (42.7)	74 (47.1)	34 (35.4)		30 (42.3)	77(42.8)	
No change	69 (27.3)	42 (26.8)	27 (28.1)		22 (31.0)	47 (26.1)	
More healthy	7 (14.6)	17 (10.8)	20 (20.8)		8 (11.3)	29 (16.1)	
Much more healthy	9 (3.6)	3 (1.9)	6 (6.3)		4 (5.6)	5 (2.8)	
**Sense of hunger and satiety**							
Much less healthy	16 (6.3)	12 (7.6)	4 (4.2)	.020	3 (4.2)	13 (7.2)	.516
Less healthy	117 (46.2)	80 (51.0)	37 (38.5)		29 (40.8)	85 (47.2)	
No change	84 (.2)	46 (29.3)	38 (39.6)		25 (35.2)	59 (32.8)	
More healthy	32 (12.6)	19 (12.1)	13 (13.5)		12 (16.9)	21 (11.7)	
Much more healthy	4 (1.6)	0	4 (4.2)		2 (2.8)	2 (1.1)	
**Amount of meals prepared at home**							
Much less healthy	13 (5.1)	10 (6.4)	3 (3.1)	.012	5 (7.0)	8 (4.4)	.144
Less healthy	56 (22.1)	38 (24.2)	18 (18.8)		18 (25.4)	37 (20.6)	
No change	84 (33.2)	59 (37.6)	25 (26.0)		29 (40.8)	55 (30.6)	
More healthy	78 (30.8)	42 (26.8)	36 (37.5)		15 (21.1)	63 (35.0)	
Much more healthy	22 (8.7)	8 (5.1)	14 (14.6)		4 (5.6)	17 (9.4)	
**Snack consumption**							
Much less healthy	30 (11.9)	21 (13.4)	9 (9.4)	.092	12 (16.9)	18 (10.0)	.321
Less healthy	121 (47.8)	79 (50.3)	42 (43.8)		31 (43.7)	87 (48.3)	
No change	74 (29.2)	45 (28.7)	29 (30.2)		21 (29.6)	53 (29.4)	
More healthy	22 (8.7)	11 (7.0)2	11 (11.5)		7 (9.9)	16 (8.9)	
Much more healthy	6 (2.4)	1 (.6)	5 (5.2)		0 (0)	6 (3.3)	
**Intake of immunity-boosting foods (such as greens, citrus fruits, etc)**							
Much less healthy	17 (6.7)	13 (8.3)	4 (4.2)	.518	4 (5.6)	13 (7.3)	.547
Less healthy	49 (19.4)	28 (17.8)	21 (21.9)		17 (23.9)	31 (17.3)	
No change	102 (40.3)	65 (34.4)	37 (38.5)		24 (33.8)	78 (43.6)	
More healthy	67 (26.5)	42 (26.8)	25 (26.0)		20 (28.2)	46 (25.7)	
Much more healthy	18 (7.1)	9 (5.7)	9 (9.4)		6 (8.5)	11 (6.1)	
**Indulging in** **more restaurant and/or fast foods**							
Much less healthy	25 (9.9)	18 (11.5)	7 (7.3)	.021	5 (7.0)	20 (11.1)	.523
Less healthy	77 (30.4)	55 (35.0)	22 (22.9)		24 (35.2)	50 (27.8)	
No change	48 (19.0)	30 (19.1)	18 (18.8)		16 (22.5)	32 (17.8)	
More healthy	45 (17.8)	19 (12.1)	26 (27.1)		11 (15.5)	34 (18.9)	
Much more healthy	58 (22.9)	35 (22.3)	23 (24.0)		14 (19.7)	44 (24.4)	
**Indulging in more unhealthy foods during moments of boredom or distress**							
Much less healthy	52 (20.6)	40 (25.5)	12 (12.5)	.014	12 (16.9)	40 (22.2)	.873
Less healthy	117 (46.2)	74 (47.1)	43 (44.8)		33 (46.5)	82 (45.6)	
No change	52 (20.6)	25 (16.6)	26 (27.1)		15 (21.1)	36 (20.0)	
More healthy	22 (8.7)	14 (8.9)	8 (8.3)		8 (11.3)	15 (8.3)	
Much more healthy	10 (4.0)	3 (1.9)	7 (7.3)		3 (4.2)	7 (3.9)	
**Number of meals eaten per day**							
Much less healthy	20 (7.9)	16 (10.2)	4 (4.2)	.257	4 (5.6)	16 (8.9)	.592
Less healthy	73 (28.9)	44 (28.0)	29 (30.2)		20 (28.2)	52 (28.9)	
No change	93 (36.8)	61 (38.9)	32 (33.3)		31 (43.7)	63 (35.0)	
More healthy	58 (22.9)	31 (19.7)	27 (28.1)		15 (21.1)	42 (23.3)	
Much more healthy	9 (3.6)	5 (3.2)	4 (4.2)		1 (1.4)	7 (3.9)	

^*^HEI is based on eight items of self-reported eating behavior changes during COVID-19 with a total combined score from 8 (much less healthy) to 40 (much healthier). The test statistics are *t* test and *X*^*2*^ test. *P*-value < .05 was considered statistically significant

Multiple regression models were computed separately by household income group since significant income group difference were identified in bivariate analysis (Table 5). Gender was included as a covariate to investigate potential associations with HEI scores in both income-based models. Gender was a nonsignificant predictor in both models. An increase in weight (Beta = -0.24, *P* = 0.004) and a big increase in weight (Beta = -0.24, *P* = 0.004), and food insecurity (Beta = -0.18, *P* = 0.023) predicted lower HEI scores for lower-income participants. Becoming less active (Beta = -0.293, *P* = 0.016) becoming much less active (Beta = -0.295, *P* = 0.014), an increase in weight (Beta = -0.243, *P* = 0.026) and food insecurity (Beta = -0.277, *P* = 0.005) predicted lower HEI scores for higher-income participants. Living with people, particularly a friend(s)/roommate(s), was a predictor of more positive healthy eating scores for lower-income participants (Beta = 0.24, *P* = 0.009) while it had no power to predict HEI scores for higher-income participants.

**Table 5:** Determinants of healthy eating scores during COVID-19 by household income, multiple regression model

**Predictors**		**Lower Income**			**Higher Income**		
		**Beta**	***P*-value**	**95% CI for Beta**	**Beta**	***P*-value**	**95% CI for Beta**
**Socio-demographics**							
Gender	Male	Ref					
	Female	-.03	.702	(-.18, .12)	-.10	.348	(-.30, .11)
Age		-.09	.355	(-.27, .10)	-.01	.932	(-.21, .20)
Student status	Not a student	Ref					
	Current student	-.07	.362	(-.14, .18)	-.06	.587	(-.27, .15)
Employment status	Not employed	Ref					
	Employed	.02	.824	(-.23, .08)	-.05	.629	(-.22, .14)
**Activity and Weight**							
Physical Activity	No change	Ref					
	Much less	-.17	.056	(–.34, .00)	-.293	.016	(-.53, -.06)
	Less	-.06	.506	(-.24, .12)	-.295	.014	(-.53, -.06)
	More	-.03	.712	(-.22, .15)	-.103	.378	(-.33, .12)
	Much more	-.03	.711	(-.19, .13)	.040	.763	(-.22, .30)
Weight	No change	Ref					
	Big decrease	.19	.015	(.10, .93)	.127	.186	(-.06, .32)
	Decrease	.04	.610	(-.12, 21)	.232	.072	(-.02, .48)
	Increase	-.24	.004	(-.41, -.08)	-.243	.026	(-.46, -.03)
	Big increase	-.24	.004	(-.40, -.07)	.090	.344	(-.10, .28)
**Food Insecurity**							
Food insecurity		-.18	.023	(-.33, -.02)	-.277	.005	(-.46, -.08)
**Social Factors**							
Live with parent(s)	No	Ref					
	Yes	.15	.145	(-.05, .35)	.09	.458	(-.15, .33))
Live with other family member(s)	No	Ref					
	Yes	-.12	.191	(-.29, .06)	-.04	.717	(-.25, .17)
Live with friend(s)/ Roommate(s)	No	Ref					
	Yes	.24	.009	(.06, .42)	.028	.821	(-.22, .27)

Dependent variable: HEI (Healthy Eating Index), *P-*value < .05 was considered statistically significant. *R*^*2*^ = .281, Adjusted *R*^*2*^ = .199 (lower income). *R*^*2*^ = .389, Adjusted *R*^*2*^ = .265 (higher income)

## Discussion

The previous lack of attention on the young adult demographic in the literature on dietary practices during the COVID-19 pandemic, aside from those that focus on college students [50], was a primary motivation for the current study. Another aim was to investigate socioeconomic and gender-based differences among young adults given the known inequities induced by the pandemic. The results of this study show a clear overall trend toward more unhealthy consumption behaviors and dietary habits of young adults during the COVID-19 pandemic while some positive changes were also found. The overall consumption of junk food significantly increased, four out of ten gained weight, and the average participant reported less healthy eating habits overall, unhealthier snack consumption, and more emotional eating. These results echo findings from other studies both in the United States and in other wealthy nations that have found associations between the pandemic and dietary deterioration [4, 12–18, 45, 50, 65]. Healthier trends included less reliance on food from restaurants, and, for some, an increase in home cooked food from scratch.

### Socioeconomic inequalities

Group level differences by household income followed a clear pattern. Lower-income participants were disproportionately represented among those who reported unhealthy consumption behaviors and dietary habits while the opposite was true for higher-income participants. Both socioeconomic groups reported more negative than positive changes. But when there were positive changes, such as less reliance on fast foods and consuming more homecooked food from scratch, higher-income participants were overly represented among those who reported them. In this way, the widening socioeconomic disparities in society at large that were caused by the COVID-19 pandemic appear to be reflected in the dietary inequities found in this study.

Socioeconomic differences may be explained, in part, by the defining characteristics of the American socioeconomic divide during COVID-19. Affluent Americans were more likely to remain employed, work from home, and not suffer income decline while lower-income Americans suffered disproportionate job loss, financial strain, and housing precariousness. Among lower-income Americans that remained working, they were more likely to work outside the home than their higher-income counterparts, and often in two or more jobs while facing risk of exposure to the virus [66, 67]. The economic impact was especially harsh for young workers who were disproportionately employed in entry level service sectors where job losses were particularly severe [52]. These disparate class-based experiences may help explain dietary inequities.

Cooking at home, for instance, requires planning, time, investment in raw products, and a modicum of stability [68], requirements that the affluent may have had enhanced capacity to meet during the pandemic and the lower-income may have had decreased capacity to meet. This study’s finding that home-based cooking from scratch significantly increased, and reliance on fast food significantly decreased among higher-income participants (but not among the lower-income) indicates that the pandemic’s positive influences on healthy eating [24, 27, 28] may have been a silver lining disproportionately enjoyed by the more privileged. On the other hand, a reliance on pre-prepared, processed, convenience and/or fast foods may have better correlated with the circumstances of those with less stable, downward spiraling lives, as they are cheap, readily available, and calorie dense. In a recent study involving focus groups, lower-income women reported that barriers to healthy eating included cost, convenience, and preparation time. This team of authors also noted that comfort foods may have been used as a coping mechanism for the multiple stressors and anxieties in their lives [69]. Another study showed a link between food insecurity and less cooking at home among low-income Americans during the pandemic [33]. In this way, the results of this study support existing research on how socioeconomic status is associated with dietary practices [70, 71].

Lower-income participants over representation among those who reported gaining weight is an added cause for concern especially since weight gain was among the strongest predictors for a lower score on the HEI. While both lower- and higher-income participants reported gaining weight during the pandemic, the proportions were more severe among those from lower-income households. When more than one in two lower-income young adults report gaining weight during the pandemic (versus one in four among higher-income participants), it suggests a class-based vulnerability to a host of metabolic diseases associated with unhealthy weight gain, such as type 2 diabetes, dyslipidemia and hypertension, all of which have been shown to downgrade immune responses, and make one more vulnerable to infections and less responsive to antivirals and vaccinations [72–74]. The Center for Disease Control and Prevention (CDC) identified obesity and increased BMI as a risk factor for COVID-19 related illnesses, regardless of age [75]. Even more disturbing is that lower-income Americans already had higher rates of obesity than their more affluent counterparts, a disparity that has long been recognized before the pandemic [76–78].

The problem of food insecurity found in this study corresponds with what is already known about the pandemic’s influence on food accessibility, and the catastrophic problems with food access, panic, hoarding, and other alarming behaviors, in the pandemic’s earliest stage [33, 79]. The fact that nearly half of lower-income participants in this study struggled with getting enough to eat at least once or twice during the first few months of the pandemic and that nearly 8% did not have enough to eat on a regular basis, is deeply concerning. This finding is supported by a previous study of college students that found that those who identified as “working class” reported higher rates of food insecurity than their middle class and affluent peers [47]. On the other hand, it should not be missed that nearly a quarter of higher-income participants reported struggling with getting enough to eat at least once or twice during the pandemic, and for more than 5%, it was a regular occurrence. It is clear, that even though income background was found to be significantly correlated with food insecurity, it did not make anyone immune from the perils of hunger and malnutrition [80].

One of the more notable findings of this study is the income-based association between living with other people and HEI scores. On the one hand, the positive influence of having people around you, coalesces with a recent study in the UK that stressed the protective buffering effect of social support from friends and family during the pandemic and other studies that found associations between social support and dietary health [81]. On the other hand, the fact that this association showed up only among lower-income participants is surprising. It could be that lower-income young adults were more susceptible to the positive influences of social support (or lack thereof) due to their more challenging socioeconomic circumstances.

### Gender inequalities

It is notable that men and women participants in this study did not significantly vary in their perceptions of healthy eating behaviors during the pandemic. But there were important gender-based nuances in how participants reported the composition of their diets which may have implications for the broader literature. For one, men’s significant increase in junk food consumption during the pandemic echoes the results of some studies that showed men’s greater intake of unhealthy products, including alcohol, during the pandemic [44, 45]. But it would seem to contradict several other studies that showed it was women who tended to eat more unhealthy foods during the pandemic [39–41], indulging more as a result of boredom, anxiety, fear, and/or depression [38, 82]. It is worth noting that in this current study, even though men reported a significant increase in junk food consumption, that it was women who reported a higher rate of eating junk food *before* the pandemic. In this way, it appears that male participants may have simply caught up with women’s previously higher rate of eating junk food while women kept indulging at a similar rate. Moreover, in respect to where participants got their food, it was both men and women who reported an increase in junk food sources.

Meanwhile, women’s overrepresentation among participants who gained weight during the pandemic reflects a globally and historically recognized problem concerning women’s over representation among the obese and severely obese [37, 78]. It lends support to other studies that have raised alarm about women’s increased vulnerability to psychological distress during the pandemic [36, 83], barriers to healthy eating including eating disorders, sleep disruptions, and fluctuations in weight [35, 48], and risks to bodily health, including cardiovascular disease [42, 43].

Future studies could investigate gender-based circumstances during the earliest stages of the pandemic to find factors that could have impacted men and women differently. While we should be particularly attentive to weight fluctuation among women, and especially weight gain, we should also strive to understand differences in reporting on issues of food insecurity and their implications. For instance, why did significantly more men in this study (10% versus 2% for women) report worrying about where their next meal was coming from? Could it be that women were more likely than men to internalize problems related to food insecurity and were therefore less likely to recognize and report it? Could it be that traditional gender roles made women more resourceful about meal sources and preparation? Could it be related to men’s greater average caloric needs compared to women? These are questions that may be better explored through a qualitative approach.

## Conclusions

This research contributes to an important body of research focused on understanding the pandemic’s uneven impacts on diet during the pandemic. With a focus on young adults, it fills a gap by addressing an age demographic that has received little attention in this literature (aside from college students). Given the way the pandemic has both exacerbated and exploited already existing disparities, it is imperative that we understand how diet and related health concerns may be reflected in them. While preventative medicine should prioritize a boosted immune system with a nutrient filled diet [84], public health policies should be especially cognizant of income-based inequities among young adults and how they reflect “differential access to the resources required to access high-quality diets” [76]. Solutions should be attentive to gender nuances and be geared toward reshaping fiscal, social and physical environments, rather than relying solely on behavioral interventions [76, 85].

### Limitations

This study had limitations. Since the data were reported retrospectively, and relied on self-reported data, it is possible that recall bias could have affected the internal reliability of the study. But since the gap in time was very small and the shift from pre-pandemic to when the pandemic began unprecedented with a very high contrast, we believe that recall bias was minimal. It should also be noted that while the study sample was relatively proportionately balanced in regard to most race and ethnic groups for this age demographic, it was under-represented by African Americans, a group that has been disproportionately harmed by the pandemic. It is possible that this under-representation could have impacted the results even though the focus of this study was not on race/ethnic differences. Also, since the majority of the participants resided in the Northeast of the United States, we were unable to capture geographical variations which could have affected our results. Finally, the relatively small sample size may caution against generalizability, though it does not diminish the importance of the results. One of the key strengths of this study is that it captured data at a pivotal moment in history which can never be collected again. The context-specific knowledge and insights of this study have illuminated important patterns and trends, and contributed to a cumulative understanding of the literature on how diets and related health behaviors were impacted by the COVID-19 pandemic. This study can serve as a foundation for future investigations whether they be qualitatively designed or involve more robust sample populations for quantitative analysis.

## Data Availability

The dataset used and analyzed during the current study are available from the corresponding author on reasonable request.

## Abbreviation

HEI: Healthy Eating Index
